# Seroprevalence of Orthopoxvirus in rural Brazil: insights into anti-OPV immunity status and its implications for emergent zoonotic OPV

**DOI:** 10.1186/s12985-016-0575-6

**Published:** 2016-07-04

**Authors:** Galileu Barbosa Costa, Lídia Teodoro Santos Augusto, Juliana Almeida Leite, Paulo César Peregrino Ferreira, Cláudio Antônio Bonjardim, Jônatas Santos Abrahão, Erna Geessien Kroon, Elizabeth Castro Moreno, Giliane de Souza Trindade

**Affiliations:** Laboratório de Vírus, Departamento de Microbiologia, Instituto de Ciências Biológicas, Universidade Federal de Minas Gerais, Belo Horizonte, Brazil; Present address: Av Presidente Antônio Carlos, 6627, Pampulha, Belo Horizonte, Minas Gerais CEP 31270-901 Brazil; Instituto Mineiro de Agropecuária, Serro, Brazil; Respiratory Virus Laboratory, Oswaldo Cruz Institute, Oswaldo Cruz Foundation (FIOCRUZ), Rio de Janeiro, Brazil; Fundação Centro de Hematologia e Hemoterapia de Minas Gerais (HEMOMINAS), Belo Horizonte, Brazil

## Abstract

**Background:**

Bovine vaccinia (BV) is a zoonosis caused by *Vaccinia virus,* a virus from *Orthopoxvirus genus (*OPV) that affects mainly cattle herds and humans in rural areas in Brazil. Because most studies have focused on outbreaks situations, data on BV epidemiology is limited. A cross sectional study in Brazilian rural areas during 2012–2013 was conducted to determine the neutralizing antibodies seroprevalence and risk factors for BV.

**Methods:**

A structured questionnaire was applied to elicit demographics data and farming practices considered risk factors for BV exposure. Neutralizing anti-OPV antibodies were investigated using plaque reduction neutralization test. The neutralizing antibodies prevalence rates were calculated and the risk factor analysis was performed using multivariate logistic regression.

**Results:**

Two hundred and forty participants were enrolled in this study with a prevalence of neutralizing antibodies of 30.8 % (95 % confidence interval [CI], 25.3–36.9). In multivariate analysis, age > 35 years (Odds Ratio [OR] = 18.2; CI 95 % = 7.7 – 43.2) and previous outbreak in property (OR = 3.9; C I95 % = 1.2 – 12.6) were independently associated with anti-OPV neutralizing antibodies.

**Conclusions:**

In this study, anti-OPV protective immunity (neutralizing antibody titers) was assessed in an endemic BV Brazilian rural area. Our findings indicate that epidemiological surveillance is required and should be applied by public health authorities to create interventions and/or prevention strategies to avoid viral spread causing future outbreaks among individuals who are under risk of infection.

## Background

*Vaccinia virus* (VACV) was used during mass vaccination against smallpox leading to its eradication [1]. Routine vaccination was ended more than 30 years ago, except for military personnel, health assistance and laboratory workers professionals in the United States and some parts of Europe [2]. Although smallpox was declared eradicated in 1980 [1], the scientific community has reassessed the level of immunity in current populations driven by the bioterrorism fear and also due to the emergence of zoonotic poxvirus around the world [3].

The emergence of zoonotic *Orthopoxvirus* (OPV) such as *Monkeypox virus* (MPXV), which is endemic in Africa [4, 5], but has also been accidentally introduced into the USA [6], *Cowpox virus* (CPXV) in Europe [7] and *Buffalopox virus* (BPXV) in India [8] has been frequently reported. Additionally, the emergence of new zoonotic OPVs such as Akhmeta virus [9], raises questions about the waning population immunity against OPV which, among other facts, could facilitate the emergence of zoonotic OPV. Nowadays, individuals less than 35 years were not vaccinated against smallpox and most of those over 35 did not receive booster immunizations since the early 70s. Therefore, immunity to smallpox and consequently to other zoonotic OPV is considered low or non-existent in the current populations [3, 10–16].

In Brazil, the emergence of Bovine vaccinia (VB), a zoonotic disease caused by VACV, is associated with rural environment and susceptible population, specially farmers/rural workers [17–19]. Domestic animals (particularly dairy cattle) and individuals who have direct contact with these animals can be affected. Nodular, ulcerated, necrotic and painful lesions are observed mainly on hands of infected people, due to contact with infected animals during the milking process [13, 17]. However, lesions can spread to secondary body sites such as forearms, arms and face [13]. Other signs and systemic symptoms have been also reported, such as fever, lymphadenopathy, headache and myalgia [13]. In general, affected people are men, predominantly aged ≤40 years, although individuals >40 years which have been vaccinated against smallpox (with presence of a vaccine take) are also affected [13, 17, 20, 21]. This fact reinforces the decrease of general immunity against OPV.

BV presents relevant economic, social and public health impacts. The burden of the outbreaks compromise hundreds of properties in all regions leading to several economic losses due to decrease in milk production and occurrence of mastitis and other secondary bacterial infections [18, 19]. The burden of this disease is stressed by the fact that Brazil has the largest commercial cattle herd in the world. Furthermore, infected individuals are unable to work due to painful lesions and overall clinical conditions, and their families, in most times, depend on their wage/income. There are high costs associated with treatment and management of infected individuals and animals, under notification of disease, misdiagnosis and the absence of a government-enforced specific surveillance policy.

This work was carried out to assess anti-OPV protective immunity of a rural population in a major dairy region in Brazil. By using this approach, only exposure that resulted in protective humoral response (ie neutralizing antibody titers) was captured. Taking into account the increasing emergence of natural OPV infections we conducted the first epidemiological study in an endemic BV area designed to understand risk factors associated with OPV infection and protective status of rural populations which are in major risk to acquire infection.

## Methods

### Study area and population

**Fig. 1:**
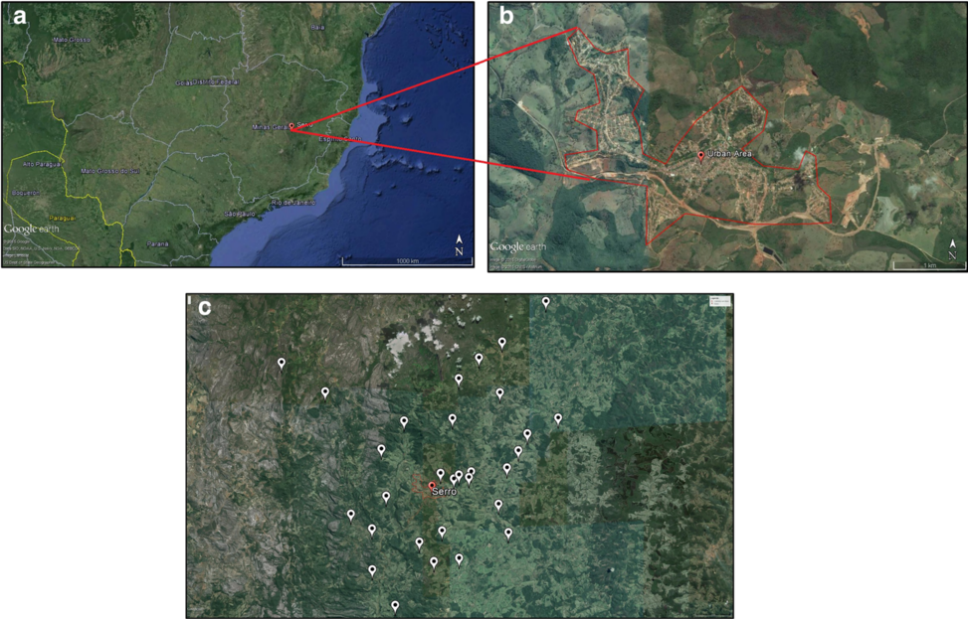
Overview of Minas Gerais State, Brazil: **a**) The point marks Serro City location in Minas Gerais State. **b** Extended view of the urban area of Serro City (marked in red). **c** Locations of the properties sampled during the course of this investigation are marked with white points (Serro outlined by the red lines). (Google Earth, 2016)

A cross sectional study was carried out from September 2012 to March 2013, using sera samples collected from people in rural areas in Serro city (18° 36’ 17” S 43° 22’ 46” W), State of Minas Gerais, Brazil (Fig. 1). Serro has a population of 20.833 inhabitants, with 7.938 residents in rural areas (1.508 farms) (IBGE, 2010).

Sample size calculation was performed using an expected prevalence of 50 % and confidence interval of 95 %, an accuracy of 10 % around the estimate and a design effect of 2.0. Using Open-Epi version 2.3.1, a minimum sample of 190 individuals was determined for the study. Considering the possibility of loss, denial and exclusion a sample size of 210 individuals was planned. However, during the field work, a total of 240 individuals were enrolled.

Monthly visits were made to dairy farms and individuals from all age groups were enrolled. The participation of individuals from small, medium and large properties was assured. Individuals who reported to live downtown and work in rural areas were also included.

### Study measures

A structured questionnaire was applied to elicit demographics data and risk factors for BV exposure. Demographic data included age, gender, self-reported skin color, occupation and education level. Questions about risk factors for BV exposure were focused on contact with cattle, horses and wild environment, practice of milking, type of milking, and consumption of raw milk and cheese. Additionally, a clinical inspection was made on left arm of participants ≥ 35 years old for the presence of smallpox vaccine scar (vaccine take), which is strongly correlated with protection, [22]. Routine smallpox vaccination was discontinued in Brazil in 1978 [1], then individuals with ≥ 35 years old should have received at least one dose of smallpox vaccine as recommended by WHO. We considered this group of individuals as vaccinated.

### Plaque reduction neutralization assay

Blood samples were collected without anticoagulants, centrifuged to harvested serum, which was stored at −20 °C until tested. A plaque reduction neutralizing test (PRNT) was used [23, 24] to assess immunological data. To this aim, sera were initially heated in a water bath at 56 °C for 30 min to denature complement system proteins and subsequently diluted in Eagle’s Minimum Essential Medium (MEM) (GIBCO®, USA) free of fetal bovine sera (FBS) to a screening ratio of 1:20. Samples were added to the same volume (1:1) of a solution containing approximately 150 plaque forming units (PFU) of VACV strain Western Reserve (WR) (VACV-WR is part of our collection and was kindly provided by Dr. C. Jungwirth, from Wurzburg University, Germany) diluted in FBS-free MEM. The final solution (virus/serum) was homogenized and incubated for 16 h at 37 °C. Six-well plates (TPP®, Switzerland) containing BSC-40 (ATCC no. CRL-2671) monolayers with approximately 90 % confluence were inoculated with virus/serum solutions; plates were incubated at 37 °C for 1 h in an atmosphere supplemented with 5 % of CO_2_. MEM supplemented with 2 % FBS (CULTILAB, Brazil) was added to each well in a volume sufficient to maintain cell monolayers during the subsequent incubation period of 48 h at 37 °C in atmosphere supplemented with 5 % of CO_2_. BSC-40 monolayers were fixed with formalin at 10 % (MERCK MILLIPORE, USA) and stained with crystal violet solution at 1 % (SYNTH®, Brazil) allowing naked eye observation of cytopathic effects. All samples were tested in triplicate. A sample was considered positive when the average number of PFUs was lower than half of the PFUs counted in the virus control (at least a 50 % reduction in PFUs). The virus control (VC), also known as the negative serum control, was made by using only FBS instead of sera; and submitted to the same protocol. It was treated as an OPV negative sample. Each six-well plate included one well reserved for the negative serum control and one for cell control (a negative control). A human serum sample obtained during a previous BV outbreak [13] was used as an OPV sera positive control.

All positive samples were titrated according to the PRNT protocol described above. Dilutions were performed in a twofold serial dilution of the sera from 1:40 to 1:5120. The last dilution in which 50 % PFU reduction was observed was used as a reference to calculate the value of neutralizing units per milliliter (NU/mL). The value was obtained by dividing 1 mL by the volume of virus/serum solution inoculated on cell monolayer and multiplying it by the least dilution that shows a 50 % reduction in PFUs.

### Statistical analysis

A descriptive analysis of the results was carried out and comparisons between those participants with and without neutralizing antibodies were made using the Pearson's chi-squared test. A *p* < 0.05 based on two-tailed alternatives was considered significant. Relative odds ratios and 95 % confidence intervals were calculated. All those variables that showed a significance level of 5 % in the univariate analysis were tested again in a multiple logistic regression model. The statistical software Epi Info^TM^ was used for the analyses.

## Results

### Demographic profile of study population

A total of 240 individuals were enrolled in this study. Demographic characteristics are presented in Table 1. The median age was 38.6 years (ranging from 5 to 90 years), being most of them over 35 years that corresponds to 52.5 %. Men represented 52.9 % of participants whereas women were 47.1 %. The majority (59.2 %) had self-reported mixed skin color.

**Table 1:** Demographic variables related to the presence of anti-OPV neutralizing antibodies

Demographics	N (%)	PRNT positive (%)	PRNT negative (%)	*p* value
Gender				
Male	127 (52.9)	44 (34.6)	83 (65.4)	0.175
Female	113 (47.1)	30 (26.5)	83 (73.5)	
Age group (years)				
>35	126 (52.5)	67 (53.1)	59 (46.9)	<0.0001
≤35	114 (47.5)	7 (6.2)	107 (93.8)	
Self-reported skin color				
Brown	142 (59.2)	44 (31.0)	98 (69.0)	0.656
Black	62 (25.8)	21 (33.9)	41 (66.1)	
White	36 (15.0)	9 (25.0)	27 (75.0)	
Education level^a^				
Elementary school or less	156 (65.0)	45 (28.9)	111 (71.1)	0.032
High school or more	60 (25.0)	16 (26.7)	44 (73.3)	
Have never gone to school	24 (10.0)	13 (54.1)	11 (45.9)	
Residence area				
Rural	208 (86.7)	63 (30.3)	145 (69.7)	0.641
Urban	32 (13.3)	11 (34.4)	21 (65.6)	
Occupation				
Rural workers	127 (52.9)	52 (21.0)	75 (59.0)	0.001
Housewives	59 (24.6)	15 (25.5)	44 (74.5)	
Others^b^	54 (22.5)	7 (13.0)	47 (87.0)	
Income^c^				
≤1 min wage	178 (74.2)	57 (32.0)	121 (68.0)	0.178
>1 min wage	19 (7.9)	9 (47.4)	10 (52.6)	
Not stated	43 (17.9)	8 (19.6)	35 (81.4)	
Total	240 (100.0)	74 (30.8)	166 (69.2)	

^a^: Elementary school or less (≤8 years of study), High school or more (>8 years of study); ^b^: this group includes other professions such as Veterinary, Zootechnician, Dentist, Seller and children; ^**c**^: income value in Brazilian currency in 2012 = R$ 622,00 (R$ 1,00 = US$ 2,08 approximately)

Most individuals had a low wage income (74.2 %) and a minimum/medium education level (65 %). The rate of illiteracy among the participants was 10 %. As expected, a high proportion of participants worked directly in rural activities (52.9 %), such as handling of domestic animals and planting. Although 22.5 % had other professions, the majority of them reported execution rural activities. Remaining individuals were housewives and children.

### Prevalence

Prevalence rates of neutralizing antibodies are presented in Table 2. Among the 240 participants, 74 showed neutralizing antibodies, representing an overall prevalence rate of 30.8 % (CI 95 % = 25.3–36.9). Antibody titers ranged from 100 to 12.800 NU/ml. The prevalence among those classified as vaccinated individuals, using the age >35 years old criterion, was 53.1 % (CI 95 % = 44.5 – 61.7), whereas 6.1 % (CI 95 % = 3.0 – 12.1) of non-vaccinated, ≤35 years old criterion, had detectable OPV neutralizing antibodies (*p* < 0.0001). If the vaccine take was used to identify the vaccinated group, the prevalence rates among those vaccinated were 57.1 % (CI 95 % = 46.0 – 67.6) and among the non-vaccinated are 18.4 % (CI 95 % = 13.2 – 25.6) (*p* < 0.0001).

**Table 2:** Prevalence rates of neutralizing antibodies in a rural population from Minas Gerais State, Brazil

Interviewed	Prevalence rate (CI 95 %)
Overall	30.8 (25.3 - 36.9)
Vaccinated^a^ (>35 years old)	53.1 (44.5 - 61.7)
Non-vaccinated^a^ (≤35 years old)	6.1 (3.0 - 12.1)
Presence of vaccine take^b^	
Yes	57.1 (46.0 - 67.6)
No	18.4 (13.2 - 25.6)

^a^: Vaccinated and non-vaccinated individuals were determined according to their age, which correspond that >35 years old participated of smallpox eradication campaign

^b^: The presence of vaccine take were determined by examination on left arm of each individual

### Potential risk factors for BV exposure

Potential risk factors for BV exposure are presented in Table 3. Among them, direct contact with domestic animals, such as bovines, equids, dogs, cats, goats and pigs was reported by 201 individuals. Overall, 70.4 % of the participants had contact with bovines and equids. However, during the visits to the properties, no typical BV lesions similar to those observed during outbreaks were seen on cattle. Almost 50 % of the participants also reported activities involving access to wild environment as hunting, gathering firewood and search for cattle and horses, which frequently run away.

**Table 3:** Exposure/risk factors related to the presence of anti-OPV neutralizing antibodies

Exposure factors	N (%)	PRNT positive (%)	PRNT negative (%)	*P* value
Contact with bovines or equids				
Yes	169 (70.4)	59 (35.0)	110 (65.0)	0.035
No	71 (29.6)	15 (21.2)	56 (78.8)	
Contact with others domestic animals^a^				
Yes	122 (50.8)	38 (25.0)	84 (75.0)	0.915
No	118 (49.2)	36 (30.5)	82 (69.5)	
Contact with wild environment^b^				
Yes	117 (48.8)	37 (31.6)	80 (68.4)	0.796
No	123 (51.2)	37 (31.8)	86 (68.2)	
Practice milking				
Yes	91 (37.9)	35 (38.5)	56 (61.5)	0.046
No	149 (62.1)	39 (26.2)	110 (73.8)	
Kind of milking^c^				
Manual	60 (65.9)	26 (43.3)	34 (56.7)	0.193
Mechanic	31 (34.1)	9 (29.1)	22 (70.9)	
Number of milkings/day^c^				
1/day	52 (57.1)	24 (46.2)	28 (53.8)	0.082
2/day	39 (42.9)	11 (28.2)	28 (71.8)	
Raw milk/cheese consumption				
Yes	217 (90.4)	70 (22.3)	147 (67.7)	0.142
No	23 (9.6)	4 (13.4)	19 (86.6)	
Participate in cheese production				
Yes	51 (21.3)	24 (47.1)	27 (52.9)	0.005
No	189 (78.7)	50 (26.5)	139 (73.5)	
Vaccine take^d^				
Yes	77 (32.1)	44 (57.1)	33 (42.9)	<0.0001
No	163 (67.9)	30 (19.4)	133 (80.6)	
Outbreak on property				
Yes	20	11 (54.0)	9 (45.0)	0.015
No	220	63 (26.9)	157 (71.3)	
Total	240 (100.0)	74 (30.8)	166 (69.2)	

^a^: Other domestic animals includes cats, dogs, goats, sheeps, pigs, chickens and ducks; ^b^: Reported by those who were in the wild environment to hunt, gather firewood and fetch some animals of property, such as horse; ^c^: The number of individuals in these two groups is relative to positives in practice milking group (*N* = 91); ^d^: Individuals who had a vaccine scar on left arm

Milking was an activity done by 91 individuals (37.9 %). In this group, most individuals practiced milking (*n* = 60; 65.9 %) only once per day (*n* = 52; 57.1 %). Consumption of unpasteurized milk and/or cheese made from this product was reported by a high proportion of interviewed individuals (90.4 %), whereas only 21.3 % were involved in the cheese*-*making process, hereby having direct contact with unpasteurized milk.

We determined a 35 years old based cut-off, representing the last vaccinated individuals before smallpox has been eradicated. Using smallpox vaccination as exposure factor, 52.5 % of interviewed individuals were vaccinated (Table 1). Indeed, a high proportion of individuals reporting to be vaccinated (*n* = 126) were considered in fact vaccinated (*n* = 77, 32.1 %), due to the presence of a vaccination scar or take on their left arm (Table 3).

### Risk factors significantly linked to higher OPV seroprevalence

The demographic characteristics significantly associated with the presence of neutralizing antibodies in the univariate analysis were age >35 years old (crude Odds Ratio = 17.3; CI 95 % = 7.4 – 40.2), illiteracy (crOR = 2.9; CI 95 % = 1.2 – 7.0), and occupation as rural workers (crOR = 2.8; CI 95 % = 1.5 – 5.1), (*p* < 0.05). Risk factors for BV exposure associated with the presence of neutralizing antibodies in the univariate analysis were contact with bovines or equids (crOR = 2.0; CI 95 % = 1.0 – 3.8), milking (crOR = 1.7; CI 95 % = 1.0 – 3.0), participation in the cheese*-*making process, (crOR = 2.4; CI 95 % = 1.3 – 4.6), have been vaccinated as measured by the presence of vaccine take (crOR = 5.9; CI 95 % = 3.2 – 10.7) and a previous outbreak in property (crOR = 3.0; CI 95 % = 1.2 – 7.7), (*p* < 0.05).

Variables that showed significant differences between those with positive and negative serology were analyzed in the multivariate logistic regression model. Variables independently associated with neutralizing antibodies were age and previous outbreak in property. Those older than 35 years were almost 20 times more likely to present neutralizing antibodies than the younger (OR = 18.2; CI 95 % = 7.7 – 43.2). Those who reported to live or work at the property at the time of previous outbreaks were almost four times more likely to present neutralizing antibodies (OR = 3.9; CI 95 % = 1.2 – 12.6) than those who did not live on the farms at that time.

## Discussion

The interruption of smallpox vaccination since 1980 has increased the susceptibility to OPV infections in human population. Nowadays, the population majority has low or non-existent immunity to OPV [3, 8, 18, 19]. With concerns about the use of smallpox virus in a bioterrorist attack, some studies have been conducted to assess immunity and protection of different populations against OPV infections [24–31]. However, natural zoonotic OPV infections which cause a burden to public health and local economy are poorly monitored worldwide, due to a gap in epidemiological surveillance.

Seroprevalence studies of OPV in different populations worldwide have also been used as a marker for the status of protection elicited by vaccination [14, 16, 23, 25, 27, 28, 30, 31]. In this study, we have assessed only anti-OPV protective immunity (ie neutralizing antibody titers) and our findings demonstrated that 53.1 % of presumed vaccinated individuals presented OPV neutralizing antibodies, in comparison to 57.1 % of true vaccinees (represented by those with vaccine take), a non-significant difference (*p* > 0.05). Similar results were observed in the United States, in studies done by Hammarlund et al. (50 %) [25] and Taub et al. (59.3 %) [30]; in Japan with individuals born between 1969 and 1975 (50 %) [27]; and in Italy (49-64 %), using ELISA IgG [8]. These findings can be explained by the fact that immunity against smallpox, and consequently other OPVs, has been believed to persist for years, as demonstrated by the studies cited above and other with military personnel [29]. Smallpox vaccination as exposure factor is also important to highlight. As in this study we found that 42.9 % of true vaccinees (33/77) are seronegative, we cannot assume that immunity is as long lasting as showed elsewhere [4, 25, 27, 28, 30]. Almost half of individuals in this category are seronegative and, indeed, neutralizing antibodies may have been maintained in the seropositive vaccinated individuals by continuous OPV exposure, since the studied region is endemic for BV [32–34]. In addition, in the studied community, the prevalence of neutralizing antibodies among the non-vaccinated individuals was 6.1 % (using the cut-off of age >35 years old) or 18.4 %, if the absence of a vaccine take was used to identify the non-vaccinated individuals. Also, it is important to highlight that all seropositive non vaccinated individuals did not present any sign of VACV infection or report to have been infected previously. This finding reinforces the possibility of the existence of alternative VACV infection routes other than the direct contact with infected cattle [34].

The OPV seroprevalence has been used as an indicator of wild OPV exposure/circulation [5, 14–16, 35, 36]. The prevalence of anti-OPV neutralizing antibodies observed in this study (30.1 %) was similar to those reported in a retrospective study performed in 2010 in Amazon region (27.9 %) [14], and higher when compared to 9.8 % found in another study done with two distinct Brazilian populations [16]. Although in these two studies [14, 16] the populations analyzed had some different characteristics and contexts for OPV serosurvey related to our study, other characteristics are similar, such as subsistence agriculture and farming as the main economic activities. Another Brazilian study carried out in the State of Rio de Janeiro showed a seropositivity of 43 % [13], where participants were from rural properties affected by outbreaks and most of them were infected, presenting clinical signs and symptoms.

Studies conducted outside Brazil, such as for BPXV in India [10], showed a higher prevalence among rural dwellers. Although the total number of individuals sampled in this study was similar to ours (*n* = 269), 121 of them were from rural areas that live in proximity with cattle and/or buffaloes, which justifies the higher seroprevalence.

Although age >35 years and previous outbreak in property were risk factors independently associated with the presence of neutralizing antibodies (*p* < 0.0001), it is impossible to distinguish if the high prevalence of neutralizing antibodies among vaccinated people was due to the background of remaining immunity from vaccinations or natural infections. On the other hand, age can be related to a higher likelihood of exposure to circulating VACV since older individuals could have had more chances to be exposed to the virus and also because a part of this population had direct contact with bovines by being farm owners or practicing manual milking. These findings are in agreement with data reported by Lederman et al. [5] who found that age > 25 years was a risk factor to OPV exposure.

As discussed by Kennedy et al. [22], vaccinated army has high titers of neutralizing antibodies when vaccinated for at least 4 years. Differently in Brazil, the population has been vaccinated only during WHO campaign, and few individuals (*n* = 13) presented high neutralizing antibodies titers (6400 – 12800 UN/ml). The high neutralizing antibodies titers found in participants >35 years and the presence of antibodies in non-vaccinated individuals reinforce the possibility of silent VACV circulation in this population.

Unexpectedly, after adjustment by all the explanatory variables, occupation, educational level, income, animal handling, consumption of raw milk or cheese, cheese production or manual milking were not associated with presence of neutralizing antibodies. The fact that most visited properties are small subsistence farms with manual milking could explain the homogeneity of risks among the studied community. A more detailed survey in an extended area, including different farming practices is necessary to better comprehend the factors involved in susceptibility and natural history of the disease in humans and animals. BV is not a mandatory notifiable disease in Brazil and there are few epidemiological data showing prevalence and risk factors in human populations.

## Conclusion

Despite the fact BV is a burden to the milk economy in Brazil, the disease is neglected and there are still few studies about seroprevalence in the country. In the present study important data regarding natural OPV circulation that is not focused on outbreaks was showed. Our results highlight the relevance of VACV virological surveillance representing a health threat with a high economical and medical burden. Furthermore, it is important to know the BV exposure risks factors in order to guide decision and recommendations to prevent future outbreaks.

## References

[1] Fenner F, Henderson DA, Arita I, Jezek Z, Ladnyi I (1988). Smallpox and its eradication.

[2] Isaacs SN (2012). Working safely with vaccinia virus: laboratory technique and review of published cases of accidental laboratory infections. Vaccinia virus and poxvirology: methods and protocols, chapter 1. Method Mol Biol.

[3] Shchelkunov SN (2013). An Increasing Danger of Zoonotic Orthopoxvirus Infections. PLoS Pathog.

[4] Rimoin AW, Kisalu N, Kebela-Ilunga B, Mukaba T, Wright LL, Formenty P (2007). Endemic human monkeypox, Democratic Republic of Congo, 2001–2004. Emerg Infect Dis.

[5] Lederman ER, Reynolds MG, Karem K, Braden Z, Learned-Orozco LA, Wassa-Wassa D (2007). Prevalence of Antibodies against Orthopoxviruses among Residents of Likouala Region, Republic of Congo: Evidence for Monkeypox Virus Exposure. Am J Trop Med Hyg.

[6] Reed KD, Melski JW, Graham MB, Regnery RL, Sotir MJ, Wegner MV, et al. The detection of monkeypox in humans in the Western Hemisphere. N Engl J Med. 2004;350:342–50. 10.1056/NEJMoa032299 14736926

[7] Essbauer S, Pfeffer M, Meyer H (2010). Zoonotic Poxviruses. Vet Microbiol.

[8] Singh RK, Balamurugan V, Bhanuprakash V, Venkatesan G, Hosamani M (2012). Emergence and Reemergence of Vaccinia-Like Viruses: Global Scenario and Perspectives. Indian J Virol.

[9] Vora NM, Li Y, Geleishvili M, Emerson GL, Khmaladze E, Maghlakelidze G (2015). Human Infection with a Zoonotic Orthopoxvirus in the Country of Georgia. N Engl J Med.

[10] Kannangai R, Finny GJ, John TJ, Sridharan G, Gopal R (2000). Vaccinia Reactive Antibodies in a South Indian Population. J Med Virol.

[11] Huhn GD, Bauer AM, Yorita K, Graham MB, Sejvar J, Likos A (2005). Clinical Characteristics of Human Monkeypox, and Risk Factors for Severe Disease. Clin Infect Dis.

[12] Karem KL, Reynolds M, Hughes C, Braden Z, Nigam P, Crotty S (2007). Monkeypox-Induced Immunity and Failure of Childhood Smallpox Vaccination To Provide Complete Protection. Clin Vaccine Immunol.

[13] Silva-Fernandes AT, Travassos CE, Ferreira JM, Abrahão JS, Rocha ES, Viana-Ferreira F (2009). Natural human infections with Vaccinia virus during bovine vaccinia outbreaks. J Clin Virol.

[14] Mota BEF, Trindade GS, Diniz TC, da Silva-Nunes M, Braga EM, Urbano-Ferreira M (2010). Seroprevalence of orthopoxvirus in an Amazonian rural village, Acre. Brazil Arch Virol.

[15] Macneil A, Abel J, Reynolds MG, Lash R, Fonnie R, Kanneh LD (2011). Serologic evidence of human orthopoxvirus infections in Sierra Leone. BMC Res Notes.

[16] Figueiredo PO, Silva-Fernandes AT, Mota BEF, Costa GB, Borges IA, Ferreira PCP (2015). Evaluating anti-Orthopoxvirus antibodies in individuals from Brazil rural areas prior to the Bovine Vaccine era. Mem Inst Oswaldo Cruz.

[17] Schatzmayr HG, Costa RVC, Gonçalves MCR, D'Andréa PS, Barth OM (2011). Human and animal infections by vaccinia-like viruses in the state of Rio de Janeiro: A novel expanding zoonisis. Vaccine.

[18] da Fonseca FG, Kroon EG, Nogueira ML, Trindade GS (2011). Zoonotic vaccinia virus outbreaks in Brazil. Future Virol.

[19] Kroon EG, Mota BEF, Abrahão JS, da Fonseca FG, de Souza Trindade G (2011). Zoonotic Brazilian vaccinia virus: from field to therapy. Antivir Res.

[20] Megid J, Appolinário CM, Langoni H, Pituco EM, Okuda LH (2008). Vaccinia Virus in Humans and Cattle in Southwest Region of São Paulo State. Brazil Am J Trop Med Hyg.

[21] Abrahão JS, Campos RK, Trindade GS (2015). Guimarães da Fonseca F, Ferreira PC, Kroon EG. Outbreak of severe zoonotic vaccinia virus infection, Southeastern Brazil. Emerg Infect Dis.

[22] Kennedy RB, Ovsyannikova IG, Pankratz VS, Vierkant RA, Jacobson RM, Ryan MA (2009). Gender effects on humoral immune responses to smallpox vaccine. Vaccine.

[23] Newman FK, Frey SE, Blevins TP, Mandava M, Bonifacio A, Yan L (2003). Improved assay to detect neutralizing antibody following vaccination with diluted or undiluted vaccinia (Dryvax) vaccine. J Clin Microbiol.

[24] Costa GB, Moreno EC, de Souza TG, Studies Group in Bovine Vaccinia (2013). Neutralizing antibodies associated with exposure factors to Orthopoxvirus in laboratory workers. Vaccine.

[25] Hammarlund E, Lewis MW, Hansen SG, Strelow LI, Nelson JA, Sexton GJ (2003). Duration of antiviral immunity after smallpox vaccination. Nat Med.

[26] Hsieh SM, Pan SC, Chen SY, Huang PF, Chang SC (2004). Age Distribution for T Cell Reactivity to Vaccinia Virus in a Healthy Population. Clin Infect Dis.

[27] Hatakeyama S, Moriya K, Saijo M, Morisawa Y, Kurane I, Koike K (2005). Persisting humoral antiviral immunity within the Japanese population after the discontinuation in 1976 of routine smallpox vaccinations. Clin Diagn Lab Immunol.

[28] Putz MM, Alberini I, Midgley CM, Manini I, Montomoli E, Smith GL (2005). Prevalence of antibodies to vaccinia virus after smallpox vaccination in Italy. J Gen Virol.

[29] Viner KM, Isaacs SN (2005). Activity of vaccinia-virus neutralizing antibody in the sera of smallpox vaccines. Microbes Infect.

[30] Taub DD, Ershler WB, Janowski M, Artz A, Key ML, McKelvey J (2008). Immunity from Smallpox Vaccine Persists for Decades: A Longitudinal Study. Am J Med.

[31] Liu Q, Huang W, Nie J, Zhu R, Gao D, Song A (2012). A Novel High-Throughput Vaccinia Virus Neutralization Assay and Preexisting Immunity in Populations from Different Geographic Regions in China. PLoS One.

[32] Trindade GS, Guedes MIC, Drumond BP, Mota BE, Abrahão JS, Lobato ZI (2009). Zoonotic Vaccinia Virus: Clinical and Immunological Characteristics in a Naturally Infected Patient. Clin Infect Dis.

[33] Assis FL, Borges IA, Ferreira PC, Bonjardim CA, Trindade GS, Lobato ZI (2012). Group 2 Vaccinia Virus. Brazil Emerg Infect Dis.

[34] Costa GB, Borges IA, Alves PA, Miranda JB, Franco-Luiz AP, Ferreira PC (2015). Alternative routes of zoonotic Vaccinia virus transmission. Brazil Emerg Infect Dis.

[35] Reynolds MG, Carroll DS, Olson VA, Hughes C, Galley J, Likos A (2010). A silent enzootic of an orthopoxvirus in Ghana, West Africa: Evidence for multi-species involvement in the absence of widespread human disease. Am J Trop Med Hyg.

[36] Reynolds MG, Damon IK (2012). Outbreaks of human monkeypox after cessation of smallpox vaccination. Trends Microbiol.

